# We are what we eat: Cross‐cultural self‐prioritization effects for food stimuli

**DOI:** 10.1111/bjop.70018

**Published:** 2025-08-13

**Authors:** Mario Dalmaso, Michele Vicovaro, Toshiki Saito, Katsumi Watanabe

**Affiliations:** ^1^ Department of Developmental and Social Psychology University of Padova Padova Italy; ^2^ Department of General Psychology University of Padova Padova Italy; ^3^ Faculty of Science and Engineering Waseda University Tokyo Japan; ^4^ Japan Society for the Promotion of Science Tokyo Japan

**Keywords:** cross‐cultural comparison, food perception, perceptual matching task, self‐prioritization effect

## Abstract

Previous research has shown that the concept of self is malleable and can be associated with various arbitrary stimuli. This study explored whether the self could be linked to images of food representative of one's own or a different culture. We compared two groups, Italian and Japanese individuals, whose cultures are both characterized by rich and distinctive food traditions. Participants performed a perceptual matching task, associating themselves with either Italian or Japanese food, depending on the block. They also reported their food habits and preferences. The findings revealed that, in both groups, the self could extend to include food stimuli from both cultural categories. However, the self was more strongly associated with food typical of the participant's own culture. Additionally, this association was unrelated to reluctance to try unfamiliar foods, as measured by the Food Neophobia Scale. These results underscore the central role of food in shaping personal identity, supporting the hypothesis of a modulatory effect of valence on the strength of self‐association with arbitrary items and suggesting that self‐related food associations may influence food preferences.

## BACKGROUND

Food plays a fundamental role in shaping both individual identities and culture. As Brillat‐Savarin famously noted, ‘*Tell me what you eat, and I will tell you who you are*’, while Feuerbach later reported a similar idea with his declaration, ‘*Man is what he eats*’. These statements effectively summarize the deep connection between food and personal identity. Indeed, food serves not only as a source of sustenance, providing the body with the necessary energy to face everyday activities, but it can also be a powerful medium for expressing traditions, values and social norms. For instance, how food is prepared, shared and consumed often reflects the historical, geographical and social aspects of a community and a country, and all these habits, which are generally learned within the core of the family, contribute to the preservation of cultural heritage. Thus, food transcends its biological function, becoming a key element in defining who we are both as individuals and as members of a cultural group.

So far, research on the influence of food on identity has primarily taken anthropological and sociological approaches, exploring how dietary practices reflect cultural values, shape social identities and promote group belonging. Key findings have emphasized the symbolic role of food in connecting individual and collective identity, the impact of ideals and social roles on food choices across ethnic groups, the way shared eating habits reinforce community bonds and the cultural differences in food meanings and their implications for well‐being (see, e.g. Devine et al., 1999; Fischler, 1988; Rozin, 2005; Sobal & Nelson, 2003). While most of these studies have relied on descriptive and observational methodologies, controlled experimental research aimed to elucidate the cognitive mechanisms underpinning the relationship between food and the construct broadly referred to as the *self* is still lacking.

The concept of self is multifaceted and, of course, comprises many different elements beyond food. For instance, it includes the body and bodily signals (e.g. our voice), the face, the physical objects we associate with (e.g. our favourite clothes), and less tangible elements such as political ideals or moral principles. These possible elements work together to shape how individuals define and understand themselves and others. Experimental evidence also reported that the self can be easily associated with entirely arbitrary stimuli, such as geometrical shapes, as documented by Sui et al. (2012). In that work, participants were first asked to associate their identity, the identity of a friend and that of a stranger, with three different geometrical shapes (e.g. circle‐self, triangle‐friend, and square‐stranger). Then, a behavioural task was administered, consisting of providing a key press to decide whether one of the three shapes appearing alongside one of three labels (i.e. ‘you’, ‘friend’ or ‘stranger’) matched or not with the previously learned information. The main results showed that participants were much faster and more accurate when the shape and the label were associated with the self (in this example, the couple ‘circle‐you’) compared to all other possible combinations. This is consistent with a self‐prioritization effect, according to which self‐related stimuli would be particularly salient and lead individuals to react to them promptly (see also Humphreys & Sui, 2016). In recent years, the work of Sui et al. (2012) has been widely replicated and extended, showing that the self can be associated with different types of stimuli, such as Gabor patches, pictures of real objects, sounds or unknown faces (e.g. Dalmaso et al., 2024; Schäfer et al., 2015, 2016; Stein et al., 2016; Woźniak et al., 2018) and exploring the impact of arbitrary self‐related stimuli on different mechanisms related to perception, attention and memory (e.g. Dalmaso et al., 2019; Martínez‐Pérez et al., 2024; Siebold et al., 2015; Stein et al., 2016; Vicovaro et al., 2024).

Of interest to the current work, a recent study (Sel et al., 2019) explored the self‐prioritization effects for food stimuli belonging to three different categories. In more detail, participants were asked to associate themselves, a friend, and a stranger with pictures of natural food (e.g. a banana), transformed food (e.g. a candy) and rotten food. Then, a matching task similar to that developed by Sui et al. (2012) was delivered, consisting of the presentation of a label (e.g. ‘you’) followed by the presentation of a food picture. The main results showed that self‐association boosted response latencies and improved accuracy, particularly for natural food items, demonstrating a more robust self‐prioritization effect for these stimuli than for transformed or rotten foods. Sel et al. (2019) proposed that food stimuli may have influenced the self‐prioritization effect by acting on attentional mechanisms, altering the allocation of attentional resources depending on the food category. The study by Sel et al. (2019) shows that the self can be linked to food stimuli, highlighting the interesting connection between identity and food and opening new avenues for research on how personal and cultural factors can shape food perception and preferences.

In this study, we explored the deep connection between food, identity and culture. Adopting a cross‐cultural perspective, we examined the self‐prioritization effect for food stimuli from two distinct cultures (Italian and Japanese) using images of typical foods from each culture as stimuli. Italian participants were tested in Experiment 1, while Japanese participants were tested in Experiment 2. Italy and Japan share key aspects of food culture, such as a focus on fresh, seasonal ingredients, balanced and nutritious meals, and the social and cultural importance of shared dining experiences. Despite the impact of globalization, individuals in both cultures continue to prefer foods rooted in their traditions (e.g. Dinu et al., 2020; Tsugane, 2021). For these reasons, the comparison between Italy and Japan provided a meaningful model to test the malleability of the self in relation to food stimuli from one's own and another culture. Consistent with the idea that food strongly influences personal and collective identity, we hypothesized a stronger association between the self and food from one's own culture compared to food from a different culture. Additionally, we explored whether individual differences in openness to unfamiliar foods could influence the strength of this association. To this end, we used the Food Neophobia Scale (FNS; Pliner & Hobden, 1992), which provides a measure of willingness to try novel or foreign foods. By integrating this measure, we aimed to investigate how food identity might be shaped by personal attitudes towards food exploration, offering new insights into the relationship between cultural identity and food selection.

## EXPERIMENT 1: ITALIAN PARTICIPANTS

### Participants

The sample size was determined a priori following guidelines for linear mixed‐effects models (Brysbaert & Stevens, 2018; see Results section), which recommend collecting at least 1,600 data points per experimental cell. Based on these guidelines, the minimum required sample size was 36 participants. However, to account for potential data exclusion, we recruited *N* = 50 (*Mean age* = 30 years, *SD* = 5.10, 29 males). All participants provided a written informed consent form, and the study was approved by the Ethics Committee for Psychological Research at the University of Padova. Participants also reported their height and weight to calculate Body Mass Index (BMI) using the formula: BMI = Weight (kg)/(Height (m))^2^. The mean BMI was 22.92 (*SE* = 0.47; *range*: 17.42–37.04). According to World Health Organization (WHO; https://www.who.int/) criteria, 39 participants were classified as normal weight, two as underweight, eight as overweight and one as obese. All participants were White Italians, recruited online via Prolific (https://www.prolific.com/).

### Stimuli, apparatus and procedure

To enhance ecological validity, we aimed to present participants with a diverse set of stimuli. Hence, we employed ten images of traditional Italian foods (bruschetta, gnocchi, grana cheese, lasagna, mozzarella, pizza, risotto, spaghetti, tiramisù and tortellini) and ten images of traditional Japanese foods (katsu curry, dorayaki, oden, okonomiyaki, onigiri, ramen, sashimi, sushi, tempura, unagi don). These images were downloaded from a generalist image database (https://www.shutterstock.com/) because, at the time of testing, no standardized databases contained both traditional Italian and Japanese foods. All images were presented at the same resolution of 400 × 267 pixels, appeared on a white background and were equalized for luminance using the SHINE_color Matlab toolbox (Dal Ben, 2023). The screen background was white and all text stimuli (font type: Hiragino Kaku Gothic Pro W3) appeared in black and were presented in Italian. Manual responses were recorded via keyboard. The experiment was programmed with PsychoPy and administered online through Pavlovia (Bridges et al., 2020). Mobile devices (i.e. smartphones and tablets) were not permitted to complete the task.

**FIGURE 1:**
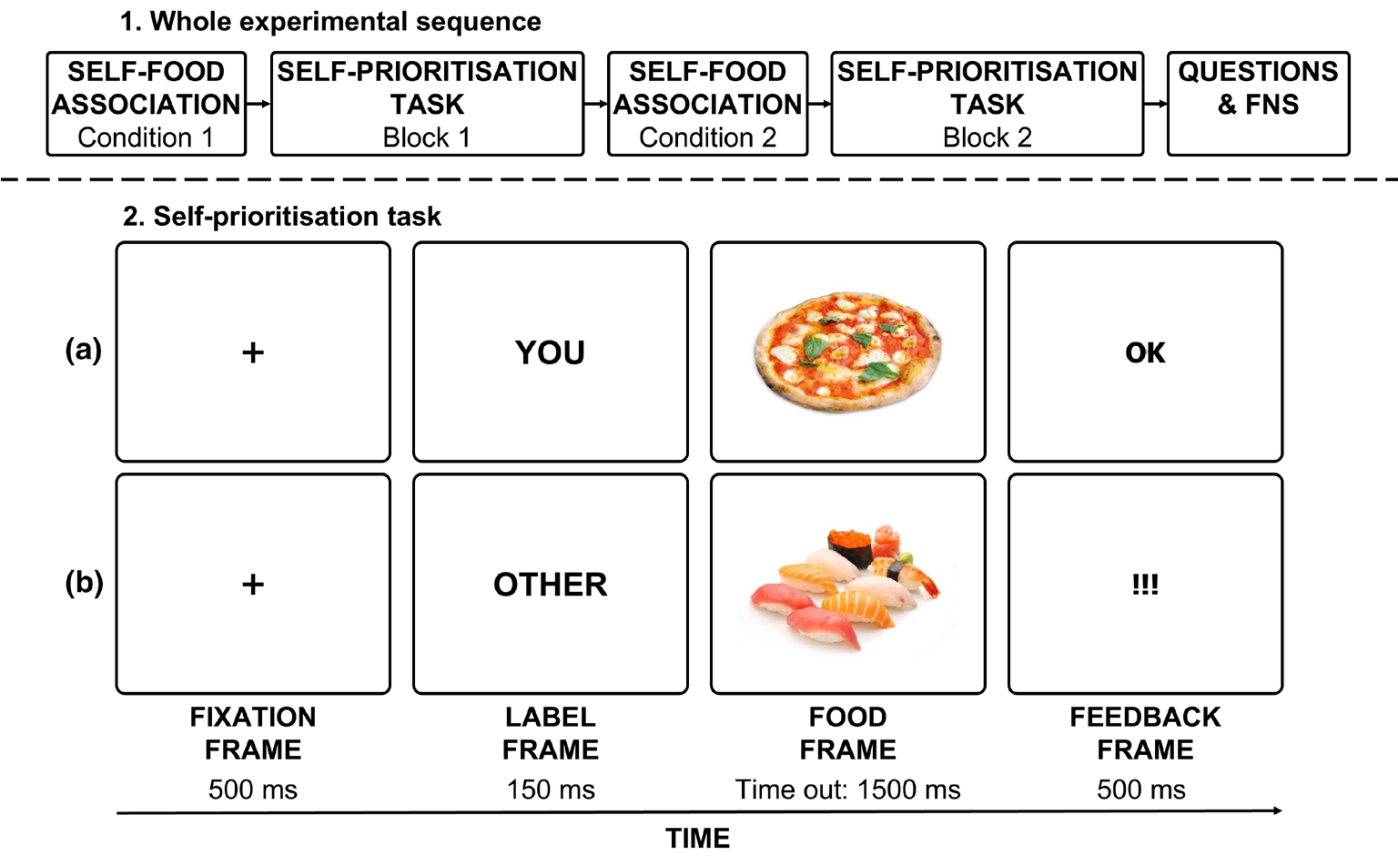
The upper section illustrates the phases of the experiment, while the lower section provides examples of trials from the perceptual matching task. In Panel a, the label ‘YOU’ is followed by an image of Italian food (in this example, pizza), with feedback indicating a correct response ‘OK’). In Panel b, the label ‘OTHER’ is followed by an image of Japanese food (in this example, sushi), with feedback indicating an incorrect response ‘!!!’). In Experiment 1, the labels ‘YOU’ and ‘OTHER’ were displayed in Italian, whereas in Experiment 2, they were presented in Japanese.

The self‐prioritization task (see Figure 1 for more details) comprised two main blocks: in one block, participants identified themselves with Italian food and another person with Japanese food; in the other block, the association was reversed. The block order was randomized across participants. The experimental procedure was similar to that used by Sel et al. (2019), who also employed food stimuli. Each block began with an initial association phase, where the participant read the following sentence, displayed centrally for 40 seconds: ‘In this experiment, you are a typical Italian food, and another person is a typical Japanese food.’ Following this, the main task began. The task started with a central black fixation cross (font‐size: 0.08 normalized units) displayed for 500 ms. Then, the word ‘YOU’ or ‘OTHER’ (font‐size: 0.08 normalized units) appeared centrally for 150 ms, followed by a centrally placed picture of a food stimulus. Participants were instructed to press a designated key (either ‘A’ or ‘L’, randomized across participants) to decide whether the word and food picture matched the previously learned identity‐food association. The picture remained visible until the participant responded or for a maximum of 1500 ms. Afterwards, central visual feedback appeared for 500 ms (the word ‘OK’ for correct responses and ‘!!!’ for incorrect or missed responses; font‐size: 0.08 normalized units).

For each condition (self‐Italian/other‐Japanese food or vice versa), participants completed a practice block of 20 trials followed by an experimental block of 180 trials, with a brief break after 90 trials. Each participant thus completed 360 experimental trials in total. The study employed a 2 (condition: self‐Italian/other‐Japanese food vs. self‐Japanese/other‐Italian food) × 2 (identity: self‐related vs. other‐related food picture) × 2 (matching judgement: matched vs. nonmatching) factorial design. The matching judgement factor referred to the pairing between the label and the food picture. For instance, when the self was associated with Italian food, matched trials included the ‘YOU’ label paired with the pizza picture or the ‘OTHER’ label paired with the sushi picture. In contrast, the reversed pairs constituted nonmatching trials. Hence, the nature of the trial (i.e. matched or nonmatching) became evident only when the food item appeared. Each experimental block (180 trials) included 45 trials per identity × matching judgement condition, with each food item appearing nine times per block. Both practice and experimental trials were presented in a random order.

Following the self‐prioritization task, participants answered several food‐related questions (see Table 1 for details). Three questions gathered information on familiarity, frequency of consumption and perceived valence for each food stimulus used in the main experiment. Each question appeared at the top of the screen 20 times, paired with one of the food stimuli presented at the centre. A response scale was displayed at the bottom of the screen, and participants provided responses at their own pace by pressing the corresponding number on the keyboard. Additionally, three questions assessed hunger levels, dietary habits and predisposition towards national food (hereafter referred to as ‘food bias’). These questions appeared once each at the centre of the screen, with a response scale below. Finally, the FNS, validated for the Italian context (Guidetti et al., 2018), was administered. The FNS included six items with a response range from 1 to 5, yielding a minimum score of 6 and a maximum of 30, with higher scores indicating greater food neophobia (see Table 1). Each FNS item appeared once at the centre of the screen with a response scale below. The whole task lasted about 30 minutes.

**TABLE 1:** From left to right: The list of the survey items organized by individual questions (1–6) and FNS item (7); the possible responses; the observed values in Italians and Japanese (mean values with standard errors in parentheses).

Survey item	Possible answers	Predicted means (SE)			
		Italian sample		Japanese sample	
		JP food	IT food	JP food	IT food
1. How familiar is this food to you?	1 = not at all, 2 = very little, 3 = a little, 4 = quite a bit, 5 = very much	3.27 (0.24)	4.80 (0.21)	4.02 (0.23)	2.38 (0.23)
2. How often do you eat this food?	1 = never, 2 = less than once a month, 3 = less than once a week, 4 = about once a week, 5 = almost every day	1.60 (0.16)	3.11 (0.17)	2.58 (0.15)	1.78 (0.15)
3. How much do you like this food?	1 = not at all, 2 = very little, 3 = a little, 4 = quite a bit, 5 = very much	3.01 (0.19)	4.38 (0.15)	4.05 (0.19)	2.92 (0.20)
4. How hungry do you feel right now?	1 = I am full, 2 = very little, 3 = a little, 4 = quite a bit, 5 = very much	3.00 (0.19)		2.30 (0.14)	
5. What is your diet?	1 = omnivorous, 2 = vegetarian, 3 = vegan, 4 = other	Frequencies omnivorous = 36; vegetarian = 4; vegan = 2; other = 2		Frequencies omnivorous = 45; vegetarian = 0; vegan = 0; other = 1	
6. How proud are you of your country's food?	1 = not at all, 2 = very little, 3 = a little, 4 = quite a bit, 5 = very much	4.27 (0.13)		4.04 (0.12)	
7. Food Neophobia Scale					
*Japanese version* A. New food‐eating experiences are important to me. B. I like to challenge myself by trying new foods. C. It is exciting to try new foods when travelling. D. Eating new food is an exciting event for me. E. I am afraid to eat things I have never had before. F. Foods from other cultures look too weird to eat. G. I don't trust new foods. H. Foods that look strange scare me.	1 = strongly disagree, 2, 3, 4 = neither disagree nor agree, 5, 6, 7 = strongly agree	Not provided		31.98 (1.24)	
*Italian version* A. I always try new and different foods. B. I don't trust new foods. C. Ethnic food seems strange. D. During the holidays, I would be willing to try new foods. E. I'm afraid of eating food I've never tried before. F. I like trying new ethnic restaurants.	1 = this phrase does not describe me at all, 2, 3, 4, 5 = this phrase describes me very much	12.27 (0.84)		Not provided	

*Note*: The first three questions (1–3) were presented on the screen alongside a picture of food. The following three questions (4–6) were shown alone on the screen. In Experiments 1 and 2, the Italian and Japanese versions of the Food Neophobia Scale (7) were used, respectively. The reported data refer to the final datasets after the removal of the responses of participants who had been classified as random responders.

### Results and discussion

#### Self‐prioritization effect

Participants who achieved less than 55% correct responses (*N* = 6) were classified as random responders and excluded from further analyses. Trials with a missing response were rare (2.82% of trials); they were discarded and not further analysed. Trials with an incorrect response (11.07% of trials) were discarded and analysed separately. Trials with a correct response and a latency smaller than 100 ms (0.10% of trials) were discarded. We considered the following factors: identity (2: you vs. other), matching judgement (2: matched vs. nonmatching) and condition (2: self_Italian_food vs. self_Japanese_food). Note that here the identity factor referred to the food picture; namely, regardless of the label shown at the start of the trial, ‘you’ trials involved a self‐related food picture, whereas ‘other’ trials involved an other‐related food picture.

**FIGURE 2:**
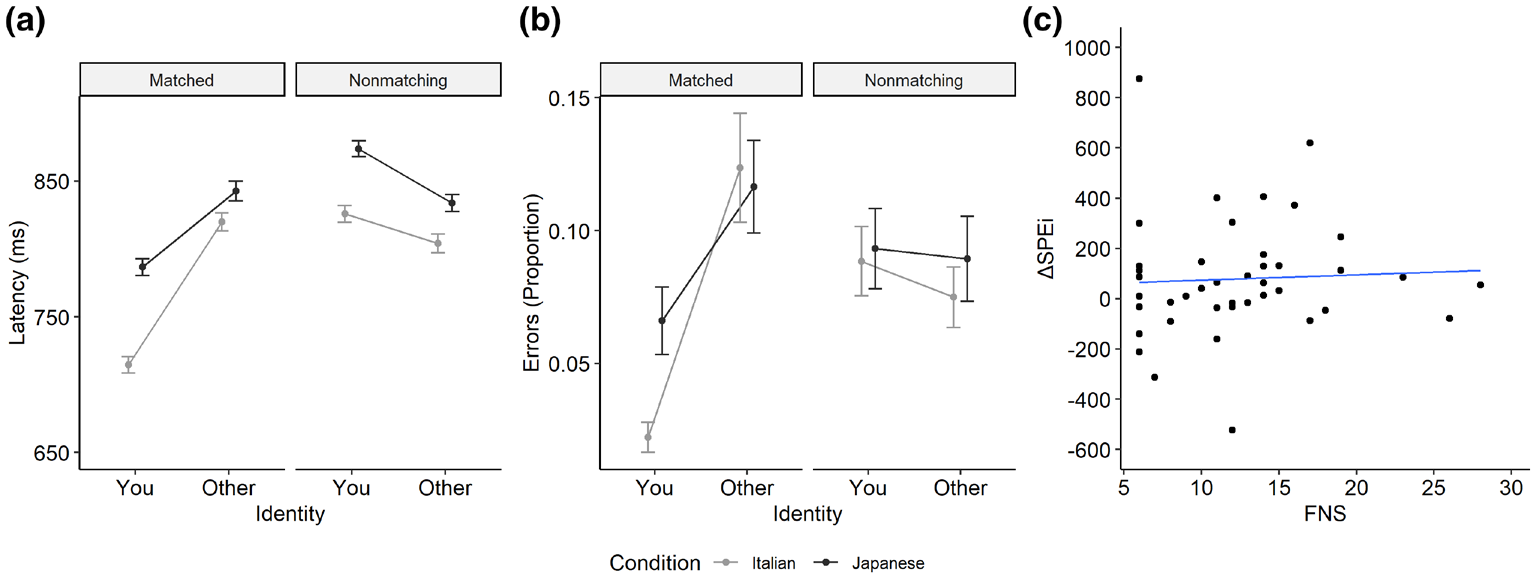
The main results of Experiment 1 (Italian participants). Panel a shows the mean latencies predicted by the best‐fitting model for latencies, while Panel b shows the proportion of correct responses predicted by the best‐fitting model for error rates, both as a function of identity and condition for matched and non‐matching trials. Error bars indicate standard errors of the mean. Panel c shows the correlation between ΔSPEi and FNS scores with a linear regression model. The represented model is based on data after the removal of three outliers with extreme ΔSPEi values.

Latencies of correctly responded trials were analysed using generalized linear mixed models (*glmer* function, R package *lme4*; Bates et al., 2015), specifying a Gamma distribution for the response variable (latency) and a Gaussian link function (Lo & Andrews, 2015). In all analyses, the fixed effects structure was held constant across models and included all main effects and interactions among the predictors (identity, matching judgement and condition). Models varied only in their random effects structure, and we selected the model with the most complete random structure among those that successfully converged (Barr et al., 2013). When multiple converging models were equally complex, model selection was based on the Akaike Information Criterion (AIC; R package *MuMIn*; Bartoń, 2023). In this specific analysis, the best‐fitting and most complex converging model included by‐subject random intercepts and slopes for the three predictors and the identity × matching judgement interaction, as well as by‐item random intercepts and slopes for matching judgement and condition. A Type 3 Wald chi‐square test was then applied to the model (R package *car*; Fox & Weisberg, 2019). The mean predicted values are graphically reported in Figure 2 (Panel a). The main effects of identity, *χ* ^2^(1) = 31.86, *p* < .001, matching judgement, *χ* ^2^(1) = 68.81, *p* < .001 and condition, *χ* ^2^(1) = 51.05, *p* < .001, were all significant. The interaction between identity and matching judgement was also significant, *χ* ^2^(1) = 219.69, *p* < .001, as well as the interaction between identity and condition, *χ* ^2^(1) = 11.91, *p* < .001. The interaction between matching judgement and condition was non‐significant, *χ* ^2^(1) = 3.07, *p* = .080. The three‐way interaction was significant, *χ* ^2^(1) = 4.73, *p =* .030, and this was further explored by analysing the data separately for matching judgement. For the matched trials, the most complete converging model had by‐subject random intercepts and slopes for identity, condition and the interaction, as well as by‐item random intercepts and slopes for condition. Both main effects were significant (*p*s < .001), as well as the interaction, *χ* ^2^(1) = 15.02, *p* < .001, revealing a stronger effect of identity when participants were associated with Italian food than with Japanese food. Specifically, pairwise comparisons (R package *emmeans*; Lenth, 2024) revealed that the difference between ‘you’ and ‘other’ trials was significant in both conditions (*p*s < .001), but its magnitude was greater when participants were associated with Italian food (106 ms) than Japanese food (55 ms). For nonmatching trials, the most complete converging model had by‐subject random intercepts and slopes for condition, as well as by‐item random intercepts and slopes for identity. The main effects of identity and condition were both significant (*ps* < .001), whereas the interaction was non‐significant (*p* = .120).^1^

Errors (coded as 1 for incorrect responses and 0 for correct responses) were analysed using the same approach as for latencies, except that the response variable was modelled using a binomial distribution with a logit link function. The best‐fitting and most complex converging model included by‐subject random intercepts and slopes for the three predictors, the identity × matching judgement interaction, the matching judgement × condition interaction, as well as by‐item random intercepts and slopes for matching judgement. The mean predicted proportions of errors are graphically reported in Figure 2 (Panel b). The main effects of identity, *χ* ^2^(1) = 36.18, *p* < .001, matching judgement, *χ* ^2^(1) = 4.78, *p* = .029 and condition, *χ* ^2^(1) = 9.56, *p* = .002, were all significant. The interaction between identity and matching judgement was significant, *χ* ^2^(1) = 46.08, *p* < .001, as well as the interactions between identity and condition, *χ* ^2^(1) = 9.76, *p* = .002, and matching judgement and condition, *χ* ^2^(1) = 7.18, *p* = .007. The three‐way interaction was significant, *χ* ^2^(1) = 15.28, *p* < .001, and this was further explored by analysing the data separately for matching judgement. For the matched trials, the most complete converging model had by‐subject random intercepts and slopes for identity, condition and the interaction, as well as by‐item random intercepts and slopes for condition. Main effects were significant (*p*s < .001), as well as the interaction, *χ* ^2^(1) = 9.46, *p* = .002, revealing a stronger effect of identity when participants were associated with Italian food than with Japanese food. Specifically, pairwise comparisons revealed that the difference between ‘you’ and ‘other’ trials was significant in both conditions, but its magnitude was greater when participants were associated with Italian (*M* = 0.098, *p* < .001) than Japanese (*M* = 0.040, *p* = .001) food. For nonmatching trials, the most complete converging model had by‐subject random intercepts and slopes for identity and condition, as well as by‐item random intercepts and slopes for identity. No significant results emerged (*p*s ≥ .558).^2^

#### Question items

Data from questions 1–3 (food stimuli) were analysed using linear mixed‐effects models, with food type (Italian food vs. Japanese food) as a fixed effect and by‐subject intercepts, by‐subject slopes and by‐item intercepts as random effects. The results revealed that Italian food, compared to Japanese food, was rated as more familiar, declared to be consumed more frequently and liked more (all *p*s < .001; see Table 1). Mean values observed for questions 4–6 (dietary behaviour) and the FNS are also presented in Table 1.

#### Correlation between the self‐prioritization effect and FNS

To explore the possible relationship between the FNS and the self‐prioritization effect, we calculated an index of the individual magnitude of the self‐prioritization effect (hereafter named ‘SPE_i_’), inspired by Sui et al. (2013). Specifically, for each participant, we computed the ratio of median RTs to the proportion of correct responses for ‘other’ trials on matching trials and the same ratio for ‘you’ trials on matching trials. The SPEi was defined as the difference between these two ratios, providing a measure directly proportional to the strength of the self‐prioritization effect. We calculated two separate SPEis for each participant: one for the self‐Italian/other‐Japanese food condition and one for the self‐Japanese/other‐Italian food condition. The difference between these two SPEis, termed ΔSPEi, represents the strength of the difference in the self‐prioritization effect when the participant's self was associated with Italian versus Japanese food. A positive ΔSPEi indicates a stronger self‐prioritization effect when associated with Italian food, whereas a negative ΔSPEi indicates a stronger effect when associated with Japanese food.

The ΔSPEi values were then correlated (Spearman's rank correlation test) with FNS scores, and a non‐significant result emerged; *r*_*s*_ = .092, *p* = .552. The results were non‐significant even after removing the data of three participants with extremely large ΔSPEi values; *r*_*s*_ = .144, *p* = .369 (see Figure 2, Panel c).

### Discussion

The main findings from Experiment 1 revealed a stronger self‐prioritization effect for Italian food stimuli compared to Japanese food stimuli. This indicates that Italian individuals associate their identities more strongly with food from their own culture, regardless of their FNS scores. In the subsequent experiment, the focus shifted to Japanese participants.

## EXPERIMENT 2: JAPANESE PARTICIPANTS

### Participants

The sample size was identical to Experiment 1. Hence, 50 participants were tested (*Mean age* = 35 years, *SD* = 4.63, 29 males). All participants provided a written informed consent form, and the study was approved by the Institutional Review Board of Waseda University. The mean BMI was 22.75 (*SE* = 0.54; *range* = 15.92–36.73). According to the WHO, 29 participants were categorized as normal weight, eight as underweight, 11 as overweight and two as obese. All participants were Asian Japanese living in Japan and were recruited online via ‘Yahoo! Crowdsourcing’ (https://crowdsourcing.yahoo.co.jp/).

### Stimuli, apparatus and procedure

All procedures were identical to those in Experiment 1, with the following exceptions: all text was presented in Japanese, and the validated Japanese version of the FNS (Kamei et al., 2024) was used. This scale included 8 items with a response range from 1 to 7, resulting in a minimum score of 8 and a maximum of 56, with higher scores indicating greater food neophobia (see Table 1).

### Results and discussion

#### Self‐prioritization effect

Data were handled and analysed as in Experiment 1. Participants who achieved less than 55% of correct responses (*N* = 4) were classified as random responders and excluded from further analyses. Trials with a missing response were rare (0.87% of trials); they were discarded and not further analysed. Trials with an incorrect response (12.72% of trials) were discarded and analysed separately. Trials with a correct response and a latency smaller than 100 ms (0.37% of trials) were discarded.

**FIGURE 3:**
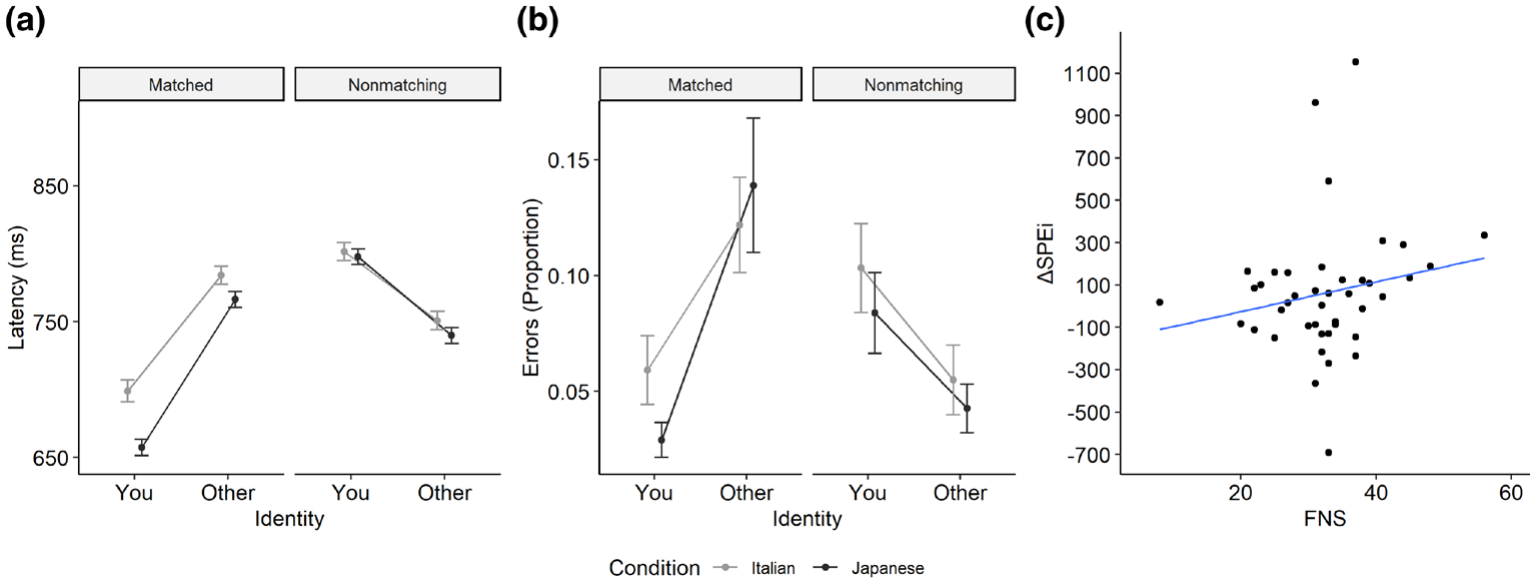
The main results of Experiment 2 (Japanese participants). Panel a shows the mean latencies predicted by the best‐fitting model for latencies, while Panel b shows the proportion of correct responses predicted by the best‐fitting model for error rates, both as a function of identity and condition for matched and nonmatching trials. Error bars indicate standard errors of the mean. Panel c shows the correlation between ΔSPEi and FNS scores with a linear regression model. The represented model is based on data after the removal of three outliers with extreme ΔSPEi values.

As for latencies, the best‐fitting and most complex converging model included by‐subject random intercepts and slopes for the three predictors and the matching judgement × condition interaction, as well as by‐item random intercepts. The mean predicted values are graphically reported in Figure 3 (Panel a). The main effects of identity, *χ* ^2^(1) = 28.32, *p* < .001, matching judgement, *χ* ^2^(1) = 81.65, *p* < .001 and condition, *χ* ^2^(1) = 10.26, *p* = .001, were all significant. The interaction between identity and matching judgement was also significant, *χ* ^2^(1) = 1216.40, *p* < .001, as well as the interaction between matching judgement and condition, *χ* ^2^(1) = 4.24, *p* = .040. However, the interaction between identity and condition was non‐significant, *χ* ^2^(1) = 0.92, *p* = .338. The three‐way interaction was significant, *χ* ^2^(1) = 12.20, *p* < .001, and this was further explored by analysing the data separately for matching judgement. For matched trials, the most complete converging model had by‐subject random intercepts and slopes for identity and by‐item random intercepts. Both main effects were significant (*p*s < .001). Despite the fact that the interaction did not reach the canonical level of significance, *χ* ^2^(1) = 3.27, *p* = .071, pairwise comparisons revealed that the difference between ‘you’ and ‘other’ trials was significant in both conditions (*p*s < .001), but the magnitude appeared to be greater when participants were associated with Japanese food (109 ms) than Italian food (83 ms). For nonmatching trials, the most complete converging model had by‐subject random intercepts and slopes for identity and by‐item random intercepts and slopes for condition. The main effects of identity and condition were both significant (*ps* < .001), whereas the interaction was non‐significant (*p* = .204). As shown in Figure 3, in nonmatching trials, ‘other’ trials showed shorter latencies than ‘you’ trials, an effect also observed in Experiment 1 (see Footnote 1 for a tentative explanation). As for errors, the best‐fitting and most complex converging model included by‐subject random intercepts and slopes for the three predictors and their interactions, as well as by‐item random intercepts and slopes for matching judgement. The mean predicted proportions of errors are graphically reported in Figure 3 (Panel b). The main effects of identity, *χ* ^2^(1) = 6.22, *p* = .013 and condition, *χ* ^2^(1) = 4.04, *p* = .045, were significant, whereas the main effect of matching judgement was not significant, *χ* ^2^(1) = 1.25, *p* = .264. The interaction between identity and matching judgement was significant, *χ* ^2^(1) = 71.0, *p* < .001. The interactions between identity and condition, *χ* ^2^(1) = 3.29, *p* = .070 and between matching judgement and condition, *χ* ^2^(1) = 0.05, *p* = .821, were non‐significant. Although the three‐way interaction did not reach the canonical level of statistical significance, *χ* ^2^(1) = 3.04, *p* = .081, we further explored the error data by analysing them separately for each level of matching judgement. To a greater extent than the statistical significance of the three‐way interaction, this approach provides a direct test of the hypothesis that the self‐prioritization effect is greater for Japanese than for Italian food. For the matched trials, the most complete converging model had by‐subject random intercepts and slopes for identity, condition and the interaction, as well as by‐item random intercepts. The main effect of identity was significant, *χ* ^2^(1) = 48.17, *p* < .001, whereas the main effect of condition was non‐significant, *χ* ^2^(1) = 2.90, *p* = .088. The interaction was significant, *χ* ^2^(1) = 6.95, *p* = .008, revealing a stronger effect of identity when participants were associated with Japanese than Italian food. Specifically, pairwise comparisons revealed that the difference between ‘you’ and ‘other’ trials was significant in both conditions, but its magnitude was greater when participants were associated with Japanese (*M* = 0.122, *p* < .001) than Italian (*M* = 0.041, *p* = .002) food. For nonmatching trials, the most complete converging model had by‐subject random intercepts and slopes for identity, condition and the interaction, as well as by‐item random intercepts. The main effect of identity was significant, *χ* ^2^(1) = 26.31, *p* < .001, whereas the main effect of condition was non‐significant, *χ* ^2^(1) = 2.49, *p* = .114. The interaction was non‐significant, *χ* ^2^(1) = 0.04, *p* = .841.^3^

#### Question items

Data were handled and analysed as in Experiment 1. Data from questions 1–3 (food stimuli) were analysed using linear mixed‐effects models, with food type (Italian food vs. Japanese food) as a fixed effect, and by‐subject intercepts, by‐subject slopes and by‐item intercepts as random effects. As expected, and in contrast to Experiment 1, the results revealed that Japanese food, compared to Italian food, was rated as more familiar, reported to be consumed more frequently and liked more (all *p*s < .001; see Table 1). Mean values observed for questions 4–6 (dietary behaviour) and the FNS are also reported in Table 1.

#### Correlation between the self‐prioritization effect and FNS

Data were handled and analysed as in Experiment 1, with the following exception. To ensure consistency in interpreting a positive ΔSPEi as indicating a stronger self‐prioritization effect for own‐culture food, the index was calculated as the difference between the SPEi in the self‐Japanese/other‐Italian food condition and the SPEi in the self‐Italian/other‐Japanese food condition. Also in this case, a non‐significant correlation emerged, *r*_*s*_ = .230, *p* = .128. The results were non‐significant even after removing the data of three participants with extreme ΔSPEi values, *r*_*s*_ = .234, *p* = .135 (see Figure 3, Panel c).

### Discussion

The main findings from Experiment 2 align with those of Experiment 1. Specifically, Japanese participants also exhibited a stronger self‐prioritization effect for food stimuli from their own culture. Furthermore, as in Experiment 1, the magnitude of this association was not related to FNS scores.

## GENERAL DISCUSSION

In this study, we investigated the self‐prioritization effect (see Sui et al., 2012) in the context of food stimuli from Italian and Japanese cultures. To this end, we conducted two separate experiments involving Italian (Experiment 1) and Japanese (Experiment 2) participants. In each experiment, participants associated themselves and a stranger with images of foods typical of the two cultures. The identity‐food association was inverted across two blocks. The main results of both experiments revealed a stronger self‐prioritization effect for foods typical of participants' own culture compared to foods from the foreign culture, regardless of their disposition towards unfamiliar foods, as assessed through the FNS.

On the one hand, the results of this study align with a broad body of evidence suggesting that the self can be associated with a wide variety of arbitrary stimuli. This malleability of the self has been demonstrated across numerous works (see, e.g. Macrae et al., 2017; Schäfer et al., 2015, 2016; Woźniak et al., 2018), which also indicated that self‐related processing can penetrate many aspects of perception and cognition. However, research in the context of food remains scarce, with only one prior study (Sel et al., 2019) directly investigating the self‐prioritization effect for different types of food stimuli (i.e. natural, transformed and rotten). The present study builds on Sel et al. (2019), confirming that the self‐prioritization effect for food is robust and generalizable to different cultures.

On the other hand, this study highlights that the strength with which individuals associate themselves with a particular stimulus can vary depending on the nature of the stimulus. In other words, our findings provide novel evidence supporting the mouldability of the magnitude of the self‐prioritization effect, as previously documented in the literature. For instance, Golubickis et al. (2021) demonstrated that the self‐prioritization effect emerged when the self was associated with preferred objects (e.g. a desirable poster) but disappeared when associated with non‐preferred objects (e.g. an undesirable poster). Similar patterns have been observed when the self was associated with faces expressing positive emotions compared to faces expressing negative emotions (Constable et al., 2021), and for symmetrical visual stimuli than asymmetrical ones (symmetry is often associated with aesthetic appeal and perceptual harmony; Vicovaro et al., 2022). Further evidence was provided by Moradi et al. (2015), who asked football fans to associate themselves with football team badges, including their preferred and rival teams. An enhanced self‐prioritization effect was observed when participants were correctly associated with the badge of their favourite team, and the strength of this effect correlated with their satisfaction with their group. Crucially, Moradi et al. (2015) also showed that this enhanced self‐prioritization effect was unlikely to be driven by stimulus familiarity. Indeed, in a control experiment, participants were asked to associate themselves with images of animals varying in familiarity (e.g. cow as highly familiar; camel as less familiar), revealing that familiarity alone did not influence matching performance. Although participants in our study reported greater familiarity with their own‐culture foods, Moradi et al.'s (2015) findings suggest that familiarity alone is unlikely to explain our results.

In general, the aforementioned studies indicate that valence would play a primary role in shaping the self‐prioritization effect. This interpretation is further supported by studies showing that the magnitude of this effect can be modulated by participants' affective state. For instance, Sui et al. (2016) reported a reduced self‐prioritization effect when participants were in a negative mood, while Hu et al. (2020) showed that associating the self with morally positive features led to stronger prioritization effects compared to associations with morally negative features. Notably, food is, by definition, a category rich in affective content and eating habits are strongly connected to emotional responses. It is therefore plausible that the enhanced self‐prioritization effect observed for own‐culture food was driven by the inherently positive valence carried by such stimuli. In addition to valence, and consistent with Sel et al. (2019), attentional processes may also have contributed to this enhancement, which also aligns with numerous studies linking this cognitive mechanism to food stimuli and individual differences, including cultural background (e.g. Sato et al., 2020). Considering the strong connection between food and survival, these results can also be viewed from an evolutionary perspective. It is plausible that the association between the self and food played a crucial role in human adaptation, closely linked to the functioning of the so‐called Behavioural Immune System (BIS; Schaller, 2011). The BIS involves psychological mechanisms designed to detect and avoid environmental pathogens, including the rejection of unfamiliar or potentially unsafe foods. Associating the self with culturally specific food items may have enhanced the ability to identify safe and nutritious options while minimizing the risk of consuming harmful or contaminated substances. This evolutionary mechanism could explain why foods of one's own culture evoke stronger self‐prioritization effects, as they are often perceived as safer and more trustworthy. Although reluctance to try unfamiliar foods, as measured by the FNS, is often considered a key component of the BIS, our findings did not reveal a significant correlation between FNS scores and the magnitude of the self‐prioritization effect. This suggests that the relationship between self‐prioritization for foods and the BIS remains unclear, at least in the present context and warrants further investigation. For instance, future studies could benefit from incorporating alternative measures that capture other dimensions of the BIS, such as scales specifically assessing sensitivity to disgust (e.g. the Disgust Scale; Haidt et al., 1994; Olatunji et al., 2007). Disgust, as a key mechanism of the BIS, may provide a complementary perspective for understanding how pathogen avoidance mechanisms influence self‐prioritization for food. In addition, the self‐prioritization effect for specific foreign foods perceived as risky or potentially unsafe could be explored. Examining how individuals associate the self with foods that differ in perceived safety or hygiene standards could provide further insights into the adaptive role of the BIS in shaping the self‐food connection.

From an anthropometric perspective, it is noteworthy that several studies have reported an influence of BMI on how individuals pay attention to and respond to food stimuli (see, e.g. Hendrikse et al., 2015, for a review). To minimize potential biases in sample selection, we decided not to recruit participants based on their BMI, assuming that BMI would reasonably fall within the normal range for most individuals, a premise supported by the descriptive statistics in both groups. Nevertheless, we conducted exploratory analyses examining the correlation between BMI and the ΔSPEi to evaluate whether BMI might have influenced our main findings. Once again, no significant results emerged (*r*_*s*_ = −.048, *p* = .654). Despite these findings, additional research could benefit from including more diverse BMI ranges and larger sample sizes, offering a more comprehensive understanding of how body‐related factors interact with self‐prioritization effects in the context of food stimuli.

This study deliberately compared two cultures with strong culinary traditions and identities, namely Italy and Japan. As already discussed, these cultures were selected because traditional culinary practices remain highly valued and persist despite globalization and increasing migration, which naturally expose people to a greater variety of food choices from different parts of the world. Future research should explore the self‐prioritization for food across a broader range of countries and cultures, including those where culinary traditions are less established or heavily influenced by dominant global cultures. For example, in cultures where local cuisines have been shaped by external influences, such as colonial history, it would be interesting to investigate whether the self‐prioritization effect is stronger for traditional foods or ‘hybrid’ options. Similarly, exploring cultures with evolving culinary identities, where traditional practices combine with modern or international influences, could shed light on how self‐related processing adapts to dynamic food contexts.

To conclude, by contributing to a growing body of research exploring the relationship between the self and culture across multiple perspectives (e.g. Golubickis et al., 2019; Jiang et al., 2019; Sparks et al., 2016; see also Markus & Kitayama, 2010), this study highlights the strong connection between self and food, showing that individuals associate more strongly with food stimuli from their own culture than those from a foreign culture. Consistently observed among Italian and Japanese participants, this finding underscores the role of food as a cultural marker and a symbolic extension of the self, shaping both personal and collective identities. These insights may have practical implications for understanding food preferences in cross‐cultural contexts, which are becoming increasingly relevant in modern societies.

## AUTHOR CONTRIBUTIONS

**Mario Dalmaso:** Conceptualization; investigation; funding acquisition; writing – original draft; methodology; validation; visualization; writing – review and editing; software; formal analysis; project administration; data curation; supervision; resources. **Michele Vicovaro:** Writing – review and editing; conceptualization; methodology; validation; visualization; formal analysis; data curation. **Toshiki Saito:** Writing – review and editing; project administration; supervision; investigation; validation; software. **Katsumi Watanabe:** Funding acquisition; writing – review and editing; project administration; resources.

## Data Availability

All data, materials and analysis scripts are available at https://doi.org/10.17605/OSF.IO/KMPX3.

## Footnotes

^1^
As shown in Figure 2, in nonmatching trials, participants showed shorter latencies for ‘other’ trials than for ‘you’ trials. Recall that ‘you’ and ‘other’ refer to the food pictures, not the labels. Thus, ‘other’ trials were those where the ‘YOU’ label was paired with an other‐related food picture. Since the label and the food picture were presented sequentially, the ‘YOU’ label may have facilitated pre‐activation of the correct food association, making participants more prepared to detect an incorrect pairing. This pre‐activation may have been weaker with the ‘OTHER’ label.

^2^
As suggested by two reviewers, we also conducted exploratory analyses including participant's sex and block order as factors. Sex did not modulate the three‐way interaction between identity, matching judgement and condition for either latencies or errors. Block order significantly modulated the three‐way interaction only for latencies, suggesting that the stronger self‐prioritization effect for Italian food was more pronounced when participants associated themselves with Italian food first (a similar pattern also emerged for errors). The R scripts for these analyses are available on OSF.

^3^
Please note that, in nonmatching trials, the lower error rate observed for ‘other’ trials aligns with the latency data (see Footnote 1 for a tentative explanation). As in Experiment 1, exploratory analyses showed that participant's sex did not modulate the three‐way interaction between identity, matching judgement and condition for either latencies or errors. Block order also did not significantly affect the three‐way interaction, although, as in Experiment 1, a trend was observed whereby the difference between the self‐prioritization effect for own‐culture food and the self‐prioritization effect for other‐culture food was stronger when participants were first associated with own‐culture food. The R scripts for these analyses are available on OSF.
